# Does an increase in visits to general practice indicate a malignancy?

**DOI:** 10.1186/s12875-016-0477-0

**Published:** 2016-07-26

**Authors:** Johannes Hauswaldt, Eva Hummers-Pradier, Wolfgang Himmel

**Affiliations:** Department of General Practice, University Medical Center, Humboldtallee 38, 37073 Göttingen, Germany

**Keywords:** Family practice, Office visits, Appointment and schedules, Cancer, Early diagnosis

## Abstract

**Background:**

An increase in a patient’s visits to doctors usually raises concerns and may be a ‘red flag’ for a patient’s deterioration of health. The aim of this study was to analyze whether an increase of patient-physician contacts is a first sign of a malignancy in a patient’s near future.

**Methods:**

This is a retrospective case-control study. From 153 German general practices’ electronic patient records (EPR), cases with at least one new malignancy diagnosis and no-malignancy controls were matched for gender and age. We calculated (1) the number of contacts in the first quarter up to the sixth quarter before a malignancy diagnosis was made and (2) the inter-contact interval (ICI), i.e. the time lag between two consecutive patient-physician contacts measured in days. Differences between cases and controls were investigated in several analyses of variance, with group and time as main factors.

**Results:**

A total of 3,310 cases and 3,310 controls could be included. The number of contacts for cases in the six quarters before a malignancy diagnosis increased from 4.8 contacts (SD 4.3) to 5.5 contacts (SD 4.8). The number of contacts for controls increased only marginally from 4.3 contacts (SD 3.6) to 4.5 (SD 4.2). The factor ‘group’ (cases vs. controls) was highly significant in the analyses of variance, also ‘time’ and the interaction ‘group * time’. The effect size, however, was very small (R^2^ being less than 0.02), which is the equivalent for about one additional contact per quarter in cases directly before a newly made malignancy diagnosis.

**Conclusion:**

An increase in contact frequency is a call for GPs to become more attentive towards these patients. It may raise the suspicion of an impending serious disease but the increase is not so dramatic and unique that it can be interpreted a reliable sign of a malignant diagnosis.

**Electronic supplementary material:**

The online version of this article (doi:10.1186/s12875-016-0477-0) contains supplementary material, which is available to authorized users.

## Background

Rapid increase in patient’s health care utilization in proximity of death is well known [1–4]. For example, in a UK case-control study in primary care, clinical features of colorectal cancer peaked out before diagnosis [5]. Studies on Danish register data also showed an increase in contacts to physicians and consumption of health care resources during one to six months before a first diagnosis of cancer was made [6, 7]. In another Danish study, the GPs’ suspicion of cancer or other serious diseases was associated with increase in a patient’s healthcare use and diagnoses of serious disease [8].

**Table:** 

*How this fits in* (1) Rapid increase in patient’s health care utilization in relation to proximity of death and of severe illness is well known. (2) On basis of German electronic patient records (EPR), we confirmed increased patient contact frequencies in primary care before a first malignancy diagnosis was made. This tendency started already several quarters before the diagnosis. However, the effect size of this trend was very small so that we cannot recommend any specific action for GPs from this. (3) Elaborated data analysis was needed for validation of results and for any specific recommendations	

Most of these studies were performed with the aim to guide health care resource allocation and communication between primary and secondary care. Another aim was to avoid delays in diagnosing cancer, attempts in the UK [9] and Denmark [10] tried to shorten and improve patient’s pathway from first signs and symptoms of cancer to diagnosis and treatment.

However, before we motivate GPs to consider every increase in visits as a first sign or ‘red flag’ of a serious disease and thus to avoid late diagnosis in cancer and referrals to specialists, we should validate former findings and determine at which time contact frequencies in case of a later cancer diagnosis increase in primary care and how strong this increase is.

Although cancer is among the leading causes of death worldwide (Global Burden of Disease 2015), incident cases of cancer are not a frequent reason for encounter in primary care. This study takes advantages of a large data set of routine data from German general practices so that enough cases can be expected to test the hypothesis that patients visit their GP more frequently several months before a malignancy is detected.

The aim of this study was to analyze whether an increase of patient-physician contacts can be observed during the six quarters (=18 months) before a diagnosis of malignancy is made and if an increase of visits is a first sign or ‘red flag’ of a malignancy in a patient’s near future.

## Method

### Study design

This is a retrospective case-control study, using routine data from electronic patient records (EPR) of 153 German general practices, covering the years between 1996 and 2006. Additional information about the database can be found in a previous report on frequency of attendance [11].

### Patients

Patients for this study were selected from anonymized raw data in two steps. First, all persons who had at least one ICD-10 malignancy diagnosis ever were identified. Second, we wanted to make sure that all of them were regular patients of the practice, and not emergency patients or patients on holiday etc. from other periods. As ‘cases’ only those were included who had at least one contact during the 18 months (540 days) before first documentation of a cancer diagnosis. A searching algorithm was developed (see Additional file 1) to identify the same number of persons matched for gender and age but without any malignancy diagnosis (‘controls’) and also having at least one visit to the practice within the six quarters prior to the quarter of first cancer diagnosis of their matched case.

The quarter in which the first diagnosis of cancer was documented was labelled q_0_. Due to characteristics of electronic databases in German primary care, we could only determine the quarter in which a patient received his or her first malignancy diagnosis, but not the exact date. This also was the reason not to include contact data from the index quarter (q_0_), as in this quarter we were unable to discern patient’s visits which occurred before a diagnosis of malignancy was made from those occurring after diagnosis.

### Calculation of contact frequencies

The main criterion of this study was the number of contacts before the malignancy diagnosis. To estimate possible differences in visits between the period immediately before and long before the malignancy diagnosis as well as possible differences in visits between cases and controls, we calculated two measures for cases and, accordingly, for controls:

1. The total number of contacts in the first quarter up to the sixth quarter before q_0_, i. e. the quarter in which a new malignancy diagnosis was first documented.
2. The inter-contact interval (ICI), i.e. the time lag between two consecutive patient-physician contacts measured in days. Lower values of ICI are equivalent to higher contact frequencies, i.e. more frequent visits. We recently showed the advantages using the inter-contact interval as a tool in analysis of health service consumption [11]. Mean inter-contact intervals were calculated for six annual quarter periods, prior to q_0_, for those patients, who had at least one contact in each of the six quarters, and compared within cases and between cases and controls.

### Statistical analysis

We first report and compare the absolute and relative frequencies of the visits in each of the six quarters before the malignancy diagnosis and in analogy for the controls. Prior to further statistical analysis, the distribution of all values of interest was checked for normal distribution using the Shapiro-Wilk test with a cut-off of *p* = 0.05, and a parallel graphical discrimination via a standardized normal probability plot (P-P plot).

The main hypothesis was to be tested in a set of two-way analyses of variance (ANOVA), with group (cases vs. controls) and time (directly before diagnosis vs. long before, i.e. one year before diagnosis) as main factors. Since it was an unbalanced design, we intended to use the type III sum of square squares to determine F and its respective *p*-value [12]. Gender was introduced as a covariate. As measures of effect, we calculated the coefficient of determination, *R*^*2*^, the non-linear correlation coefficient eta-squared, and partial eta-squared. Additionally, an analysis of variance with contact frequencies as repeated measurements [13] was used for a subsample of cases and controls who visited their GP at least once in each of the last six quarters prior to q_0_.

If the values of interest were not normally distributed the scaling of the variables was to be modified by an adequate transformation, e. g. a log transformation.

Stata 12 was used for all statistical calculations and graphical display.

## Results

### The sample

A total of 3,310 patients in our sample had at least one malignancy diagnosis and at least one practice visit within the six quarters (540 days) prior to the first day of the index quarter of their first new malignancy diagnosis. These ‘cases’ were matched with 1,485 male and 1,825 female patients most similar with respect to age (‘controls’). To gain these 3,310 control matches, all patients from the raw sample without any malignancy diagnosis ever had to be run 89 times through a generic identification algorithm (see Additional file 1).

### Contact frequencies

**Fig. 1:**
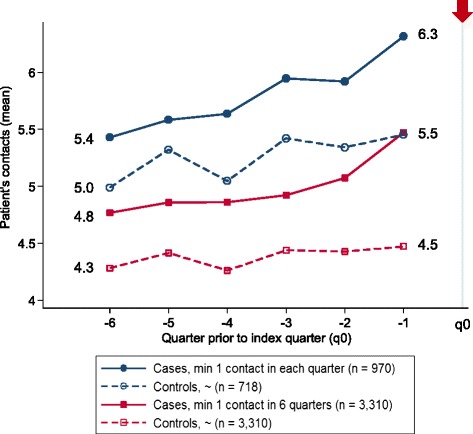
Mean contact frequency, six annual quarters

Figure 1 shows the mean number of patient contacts per quarter, separated for cases and controls and separated for all patients vs. a subsample of those who visited their GP at least once in each of the last six quarters prior to the index quarter. This subsample consisted of 718 controls, with 429 females (59.8 %), and 970 cases, with 569 females (58.7 %).

The number of visits for cases in the complete sample increased, on average, from 4.8 contacts (SD 4.3) to 5.5 contacts (SD 4.8) per quarter; the number of contacts for controls increased, on average, only marginally from 4.3 contacts (SD 3.6) to 4.5 (SD 4.2). The result was similar for those who visited their GP at least once in each of the last six quarters (subsample), with a considerable increase for cases from 5.4 (SD 4.5) to 6.3 (SD 4.7) and a slight increase for controls from 5.0 (SD 3.8) to 5.5 (SD 4.7) per quarter.

### Changes in inter-contact intervals (ICI)

In the next step, we compared the ICI over time and between cases and controls. The ICI for controls remained nearly stabile during the whole study period. For example, controls had, on average, an ICI of 52.5 days (SD: 101.4; median: 25.5) in the sixth quarter before the index one, and it was nearly the same in the quarter prior to the index one (53.0; SD: 105.0; median: 26). In contrast, the average ICI for cases was 45.3 days in the sixth quarter (SD: 94.3; median: 24.5) and decreased to 41.3 days (SD: 84.5; median: 20.5) in the quarter directly before the diagnosis. In in other words, not only visited these patients their GP in shorter intervals just before they received a malignancy diagnosis; compared to their matched cases, they also visited their GPs in shorter intervals already six quarters before diagnosis.

### Analysis of variance in contact frequencies

Since our main criterion, visits per quarter, was not normally distributed, we log-transformed the values and then performed a two-way analysis of variance with the main factors ‘group’ (cases vs. controls) and ‘time’. Gender was introduced as a covariate. When comparing the first vs. the second quarter (Table 1), the factor 'group' was significant with an impressive F value (*F* = 102.6; *p* < 0.0001). When comparing the first vs. the sixth quarter (Table 2), the factor 'group' was still significant, with a high F value (59.9). ‘Time’ and the interaction ‘group * time’ were also significant, but only when comparing first quarter vs. sixth quarter (Table 2). However, the effect size in both analyses of variance was very small, with *R*^*2*^, the population squared multiple correlation, of 0.011 and 0.012, respectively and an eta-squared, the overall non-linear correlation coefficient, of 0.01 for both. Accordingly, the effect sizes for all independent factors, the partial eta-squared values, were also smaller than 0.01.

**Table 1:** ANOVA, contacts in quarter-2 vs. quarter-1

Source	df	Mean square	F statistic	Pr > F
Model	4	3.83	27.91	< 0.0001
Error	9,377	0.14		
Gender	1	0.07	0.50	0.4773
Case/control	1	14.08	102.55	< 0.0001
Quarter	1	0.30	2.21	0.1376
Case/control*Quarter	1	0.43	3.12	0.0775

3,310 cases and 3,310 controls

Dependent variable: Number of contacts per quarter, logarithm

Effect size adjusted R^2^ = 0.0113

* linking two independent variables is to indicate their interaction term included in the ANOVA modelling equation

### Analysis of contact frequencies over six quarters as repeated measures

We performed a further analysis of variance with contact frequencies during the six quarters before q_0_ as repeated measures for those patients only who had consulted their GP in each of the six quarters under study. Table 3 shows the 'between effect', with 'group' being significant (*F* = 8.4; *p* < 0.0038). Table 4 shows the 'within effect', with 'time' being significant and a rather high *F* value (*F* = 12.7, *p* <0.0001) together with a slight interaction with group. But the effect sizes were, again, extremely small (Tables 3 and 4).

**Table 2:** ANOVA, contacts in quarter-6 vs. quarter-1

Source	df	Mean square	F statistic	Pr > F
Model	4	3.24	24.19	< 0,0001
Error	7,852	0.31		
Gender	1	0.10	0.77	0,3787
Case/control	1	7.95	59.32	< 0,0001
Quarter	1	0.86	6.38	0,0115
Case/control*Quarter	1	1.20	8.95	0,0028

3,310 cases and 3,310 controls

Dependent variable: Number of contacts per quarter, logarithm

Effect size adjusted R^2^ = 0,0117

* linking two independent variables is to indicate their interaction term included in the ANOVA modelling equation

**Table 3:** Repeated-measures ANOVA, 6 quarters: between-subject effects

Source	df	Mean square	F statistic	Pr > F
Gender	1	0.01	0.00	0.9903
Case/control	1	608.1	8.39	0.0038
Gender*Case/control	1	103.09	1.42	0.2335
Error (contacts)	1,684	72.57		

970 cases and 718 controls

Dependent variable: number of contacts per quarter, logarithm

Effect size R^2^ = < 0.01

* linking two independent variables is to indicate their interaction term included in the ANOVA modelling equation

**Table 4:** Repeated-measures ANOVA, 6 quarters: within-subject effects

Source	df	Mean square	F statistic	Pr > F
Contacts	5	102.23	12.72	< 0.0001
Contacts*Gender	5	4.75	0.59	0.7067
Contacts*Case/control	5	19.15	2.38	0.0361
Contacts*Gender*Case/control	5	12.06	1.5	0.1861
Error (contacts)	8,420	8.04		

970 cases and 718 controls

Dependent variable: number of contacts per quarter, logarithm

Effect size R^2^ = < 0.01

* linking two independent variables is to indicate their interaction term included in the ANOVA modelling equation

## Discussion

### Summary of main findings

Patients visited their GP more often during the time period immediately before a first malignancy diagnosis was made, compared to earlier periods. This is the result of a retrospective observational study, incorporating routine data from German general practices’ EPR into a case-control design. The changes over time as well as the differences between cases and controls were highly significant but the effects were not very impressive, with an average increase of about 1 contact per quarter or, in terms of inter-contact intervals, a decrease of about six days between two consecutive consultations.

### Strengths and limitations of the study

The results of this study are based on a large sample of primary care patients whose visits are well-documented because data are also used for billing and reimbursement of medical services. Due to the retrospective character of the study, the behavior of both patients and GPs is not influenced by any observational effects so that the visit behavior mirrors real world primary care, being at the same time unobtrusive.

The study sample is a convenience sample from routine data of general practices located in different parts of Germany and is covering the time period from 1996 to 2006. This sample, which has been used in prior studies [11, 14], cannot be considered to be representative for all German GPs.

Patient’s contacts to the GP were derived from fee-for-service data and other information in this rich and comprehensive routine data sample. Nevertheless, there may have been additional visits which were not recorded in these EPR service data, so calculation of inter-contact intervals (ICI) and contact frequencies may err on the conservative side [14].

First diagnosis of any malignancy was taken from first appearance of respective ICD code, from chapter 2 (“Neoplasms”). We did not stratify the sample according to entities. We did not extend our studies for first appearance of any other serious disease than malignancy. For this last reason, again our results may err on the conservative side, as other serious reasons may induce a patient’s increased visits with the GP as well but this patient has been subsumed under the controls.

Routine data from German practice information systems yield confirmed ICD codes on quarter of a year base only. For this reason we used the annual quarter of a patient’s first malignancy detection as a proxy for referencing contacts and inter-contact intervals. This may have caused a slight bias and conservative error, as for this index quarter (q_0_) we were unable to discern patient’s contacts which occurred before a diagnosis of malignancy was made from those occurring after diagnosis and for this reason had to exclude completely contact data from this quarter.

German laws on privacy protection and data security grossly limit secondary use and analysis of primary data from routine health care, in contrast to many other countries, for example Denmark with a comprehensive national register [15], or the United Kingdom with a primary care data base derived from routinely recorded electronic patient records [16]. In spite of these regulations, we received ethical approval to gain pseudonymized routine data for the years 2001 to 2003 and 2006 to 2007 via a mandatory software interface from electronic patient records of more than 160 general practices in Germany. These data are the base of this study. We are optimistic that we will have access to new data in two or three years but so long our data are the only ones available to perform such an analysis. It seems to be reasonable to assume that the main results of our study-namely that visits increase significantly over the quarters directly before a malignancy diagnosis is made, but effect size is rather small-could be confirmed if the analysis is repeated with data from a more recent period of observation.

### Comparison with literature

Our analysis from GPs’ routine data showed an increase in visits of patients to their GP prior to first malignancy diagnosis. While population-based case-control studies from the UK [5] and Denmark [6, 7] showed similar trends shortly before the diagnosis, our study demonstrates that these trends can be observed in primary care practices even longer times before, at least for some patients.

Although the modelled differences were highly significant in our sample, the effect size of the increase in contact frequencies was very small and far less impressive than the fourfold up to tenfold increase [5], or even more [6] reported in other studies. According to Christensen et al. [7], cancer patients had twice as many consultations with their general practitioner (GP) 3 months before their diagnosis, ten to eleven times more diagnostic investigations and five times more hospital contacts than the reference population. Here the demand for GP services peaked 1 month before diagnosis. One reason for our lower rate of increase may be the use of a single type primary data source, in contrast to the cumulative data and parameter acquisition by the other studies, thus for example missing those rapidly or seriously ill patients who can by-pass their GP and enter secondary and specialist care rather easily in Germany [17]. Another reason may be that German patients in general have a high annual base rate in visiting their GP when compared to patients from other countries, with about ten consultations per capita in 2012 in Germany, compared to about five consultations in the UK or Denmark [18].

In contrast to other studies (e.g. [3, 4, 7]) which considered several outcomes, such as contacts and resources, our analysis was limited to contacts only since we wanted to recognize whether visits of patients prior to malignancy diagnosis start to increase at a certain point of time. However, this could not be determined by our study. Rather, a uniform increase happened over the six quarters under study, and possibly before. This was also confirmed by our analyses of variance where the interaction of time and group (cases vs. controls) was only significant when we compared the first vs. the sixth quarter before diagnosis of a malignancy but not when comparing the first vs. the second quarter. Consequently, time was highly significant in the analysis of variance with contact frequencies as repeated measurements over six quarters. In other words, our study confirmed an increase in visits of cancer or pre-cancer patients but this seems to be a gradually incrementing development, on average, and not a sudden step-like increase some weeks or months before a definite diagnosis was made.

### Implications for practice and future research

Every physician should and will be alerted when a known patient visits him more frequently than is to be expected from the patient’s own prior history. Observing this may arouse suspicion that a serious unknown medical problem may be the cause for a change in consultation behavior. And it may be attractive to formulate our preliminary findings as a rule: If a patient’s average contact frequency derived from his last six (or more) visits to the GP is higher than the average from the same amount of preceding visits, the GP may expect a diagnosis of malignancy in this patient. But this rule would be far too simple and, what is even more important, dangerous. The increase in visits is not so dramatic that it could help to distinguish an alarming contact behavior from a random increase, whatever the reason may be. Richards [9] described a similar dilemma from the National Awareness and Early Diagnosis Initiative, considering the many symptoms that could possibly be due to cancer. At which point should a GP reassure, observe, request investigations or refer to specialist services? In other words, the measure-be it contact frequencies or symptoms-is not specific enough, may raise “false alarm” and cause over-diagnosis when labelling patients as cancer or pre-cancer candidates without sufficient evidence.

This is unsatisfactory since there seems to be, indeed, an increase in contact frequencies and contact intervals in pre-cancer patients or patients with an unknown malignancy diagnosis. Future research should find out whether there are any additional characteristics in consultation behavior or socio-demographic data of patients that make the detection of a cancer candidate more specific without alarming other patients. So far, the GP’s gut feeling remains a strong predictor of cancer, as a Danish study found out [10] and the observation of increased contact frequencies may support this gut feeling.

## Conclusion

An increase in contact frequency before a malignancy diagnosis is a call for GPs to become more attentive towards these patients. However, the increase is not so dramatic and unique that it can be interpreted as a reliable sign of a new malignancy diagnosis and does not require, as a general rule, any specific action from GPs.

## Additional file

Additional file 1: (Searching algorithm for matched controls’ identification). (PDF 21 kb)
