# Supplementary material for: Does an increase in visits to general practice indicate a malignancy?
Source: BMC Fam Pract. 2016 Jul 26;17:94. doi: 10.1186/s12875-016-0477-0 (PMC4960682; doi:10.1186/s12875-016-0477-0)
Supplement: Additional file 1: — (Searching algorithm for matched controls’ identification). (PDF 21 kb) [file 12875_2016_477_MOESM1_ESM.pdf]

Appendix A

(Searching algorithm for matched controls' identification)

```
* This do-file finds controls (non-cancer patients) matching in gender and age
* to 3,310 cases (cancer patients) which were indexed as having
* at least one practice contact within the 6 quarters prior to the quarter of
* first cancer diagnosis (q0).
* The controls, matched 1:1, also have to fulfill the condition of having
* at least one practice contact within the 6 quarters prior to the quarter of
* first cancer diagnosis (q0) of their matched case.
*
*
* For do-file development, reset studypat and studpatfinal to starting condition
*
*use "D:\EigDatNotebook20130901\2 RoutinDatNutzg\ICI 2014\ICIearlyCaDx\ICIearlyCaDx data\study data\studydata_v15\studypat_20150331_clone0.dta", clear
*save "D:\EigDatNotebook20130901\2 RoutinDatNutzg\ICI 2014\ICIearlyCaDx\ICIearlyCaDx data\study data\studydata_v15\studypat_20150331_clone.dta", replace
*use "D:\EigDatNotebook20130901\2 RoutinDatNutzg\ICI 2014\ICIearlyCaDx\ICIearlyCaDx data\study data\studydata_v15\studypatfinal\studypatfinal_20150331_clone0.dta", clear
*save "D:\EigDatNotebook20130901\2 RoutinDatNutzg\ICI 2014\ICIearlyCaDx\ICIearlyCaDx data\study data\studydata_v15\studypatfinal\studypatfinal_20150331_clone.dta", replace
*
* Find nearest control to unmatched case, attribute case's q0 and id_pair540, and flag both as provisionally matched
*
use "D:\EigDatNotebook20130901\2 RoutinDatNutzg\ICI 2014\ICIearlyCaDx\ICIearlyCaDx data\study data\studydata_v15\studypat_20150331_clone.dta", clear
gsort id_sort
replace q0=q0[_n+1] if ( ( ind_chap2==0) & ( q0==0) & ( id_pair540==.) & ( ind_matchalready==0) & ( ind_chap2[_n+1]==1) & ( q0[_n+1]>0) & ( id_pair540[_n+1]>0) & ( id_pair540[_n+1]<.) & (ind_matchalready[_n+1]==0))
replace id_pair540=id_pair540[_n+1] if ( ( ind_chap2==0) & ( q0== q0[_n+1]) & ( id_pair540==.) & ( ind_matchalready==0) & ( ind_chap2[_n+1]==1) & ( q0[_n+1]>0) & ( id_pair540[_n+1]>0) & (id_pair540[_n+1]<.) & ( ind_matchalready[_n+1]==0))
replace ind_matchalready=1 if ( ( ind_chap2==0) & ( q0==q0[_n+1]) & ( id_pair540== id_pair540[_n+1] ) & ( ind_matchalready==0) & ( ind_chap2[_n+1]==1) & ( q0[_n+1]>0) & ( id_pair540[_n+1]>0) & ( id_pair540[_n+1]<.) & ( ind_matchalready[_n+1]==0))
replace ind_matchalready=1 if ( ( ind_chap2==1) & ( q0==q0[_n-1]) & ( id_pair540== id_pair540[_n-1] ) & ( ind_matchalready==0) & ( ind_chap2[_n-1]==0) & ( q0[_n-1]>0) & ( id_pair540[_n-1]>0) &  ( ind_matchalready[_n-1]==1))
save "D:\EigDatNotebook20130901\2 RoutinDatNutzg\ICI 2014\ICIearlyCaDx\ICIearlyCaDx data\study data\studydata_v15\studypat_20150331_clone.dta", replace
*
* Write / flag matches into studydata
*
use "D:\EigDatNotebook20130901\2 RoutinDatNutzg\ICI 2014\ICIearlyCaDx\ICIearlyCaDx data\study data\studydata_v15\studydata_20150331_clone.dta", clear
merge m:1 id_patient using "D:\EigDatNotebook20130901\2 RoutinDatNutzg\ICI 2014\ICIearlyCaDx\ICIearlyCaDx data\study data\studydata_v15\studypat_20150331_clone.dta", update replace
*
* Flag non-fitting control
*
replace ind_matchalready=-1 if ( ( ind_chap2==0) & (( q0>0) & ((( q_date-q0)<-6) | (( q_date-q0)>-1))))
collapse (max) ind_matchalready, by ( id_patient)
save "D:\EigDatNotebook20130901\2 RoutinDatNutzg\ICI 2014\ICIearlyCaDx\ICIearlyCaDx data\resulting data\20150331_backfire_clone.dta", replace
*
* Write back into list of studypat, save matched pairs found, delete non-fitting controls, and include unmatched cases into search again
*
use "D:\EigDatNotebook20130901\2 RoutinDatNutzg\ICI 2014\ICIearlyCaDx\ICIearlyCaDx data\study data\studydata_v15\studypat_20150331_clone.dta", clear
merge 1:1 id_patient using "D:\EigDatNotebook20130901\2 RoutinDatNutzg\ICI 2014\ICIearlyCaDx\ICIearlyCaDx data\resulting data\20150331_backfire_clone.dta", update replace
drop _merge
gsort id_sort
replace ind_matchalready=0 if ( ( ind_chap2==1) & ( q0==q0[_n-1]) & ( id_pair540== id_pair540[_n-1] ) & ( ind_matchalready==1) & ( ind_chap2[_n-1]==0) &  ( ind_matchalready[_n-1]==-1))
save "D:\EigDatNotebook20130901\2 RoutinDatNutzg\ICI 2014\ICIearlyCaDx\ICIearlyCaDx data\study data\studydata_v15\studypat_20150331_clone.dta", replace
*
use "D:\EigDatNotebook20130901\2 RoutinDatNutzg\ICI 2014\ICIearlyCaDx\ICIearlyCaDx
```

```
data\study data\studydata_v15\studypat_20150331_clone.dta", clear
gsort id_sort
drop if ( ind_matchalready != 1)
gsort id_sort
append using "D:\EigDatNotebook20130901\2 RoutinDatNutzg\ICI
2014\ICIearlyCaDx\ICIearlyCaDx data\study
data\studydata_v15\studypatfinal\studypatfinal_20150331_clone.dta"
save "D:\EigDatNotebook20130901\2 RoutinDatNutzg\ICI 2014\ICIearlyCaDx\ICIearlyCaDx
data\study data\studydata_v15\studypatfinal\studypatfinal_20150331_clone.dta", replace
*
use "D:\EigDatNotebook20130901\2 RoutinDatNutzg\ICI 2014\ICIearlyCaDx\ICIearlyCaDx
data\study data\studydata_v15\studypat_20150331_clone.dta", clear
gsort id_sort
drop if ( ind_matchalready==1)
drop if ( ind_matchalready==-1)
save "D:\EigDatNotebook20130901\2 RoutinDatNutzg\ICI 2014\ICIearlyCaDx\ICIearlyCaDx
data\study data\studydata_v15\studypat_20150331_clone.dta", replace
*
* End of do-file
```
